# Sustained Attention and Working Memory Predict the Number of Days on Health-Related Benefits in the Year Following Occupational Rehabilitation

**DOI:** 10.1007/s10926-020-09944-5

**Published:** 2021-01-20

**Authors:** Thomas Johansen, Irene Øyeflaten, Hege R. Eriksen, Peter S. Lyby, Winand H. Dittrich, Inge Holsen, Hanne Jakobsen, Ruby Del Risco Kollerud, Chris Jensen

**Affiliations:** 1 Norwegian National Advisory Unit on Occupational Rehabilitation, Haddlandsvegen 20, 3864 Rauland, Norway; 2 NORCE, Norwegian Research Centre, Bergen, Norway; 3 Department of Sport, Food and Natural Sciences, Western Norway University of Applied Sciences, Bergen, Norway; 4 Catosenteret Rehabilitation Center, Son, Norway; 5 FOM Hochschule, KCI Competence Center for Behavioral Economics, Frankfurt, Germany; 6 Red Cross Haugland Rehabilitation Center, Flekke, Norway; 7 Valnesfjord Health Sports Centre, Fauske, Norway

**Keywords:** Occupational rehabilitation, Return to work, Sick leave, Cognition, Attention, Memory

## Abstract

*Purpose* The objective of this study was to investigate the association between cognitive and emotional functioning and the number of days on health-related benefits such as sick leave, work assessment allowance and disability pension. We investigated whether cognitive and emotional functioning at the start of rehabilitation and the change from the start to the end of rehabilitation predicted the number of days on health-related benefits in the year after occupational rehabilitation. *Methods* A sample of 317 individuals (age 19–67 years), mainly diagnosed with a musculoskeletal or mental and behavioural ICD-10 disorder, participated. The sample was stratified depending on the benefit status in the year before rehabilitation. Those receiving health-related benefits for the full year comprised the work assessment allowance and disability pension (WAA) group and those receiving benefits for less than a year comprised the sick leave (SL) group. The participants were administered cognitive and emotional computerised tests and work and health questionnaires at the beginning and end of rehabilitation. The cumulative number of days on health-related benefits during 12 months after rehabilitation was the primary outcome variable and age, gender, educational level, subjective health complaints, anxiety, and depression were controlled for in multiple regression analyses. *Results* The WAA group (n = 179) was significantly impaired at baseline compared to the SL group (n = 135) in focused attention and executive function, and they also scored worse on work and health related variables. Higher baseline scores and change scores from the start to the end of rehabilitation, for sustained attention, were associated with fewer number of health-related benefit days in the WAA group, while higher baseline scores for working memory were associated with fewer number of health-related benefit days in the SL group. *Conclusions* New knowledge about attention and memory and return to work in individuals with different benefit status may pave the way for more targeted programme interventions. Rehabilitation programmes could benefit from designing interventions that respectively improve sustain attention and working memory related to working life in individuals on sick leave or work assessment allowance and disability pension.

## Introduction

Participation in working life involves mental and cognitive demands, coping with different social interactions, adjusting to multiple roles, and adapting to various occupational contexts. Therefore, it can be assumed that cognitive and emotional abilities such as attention, memory, executive function and emotion regulation are essential for performing well in working life [1–3]. Cognitive impairments are prevalent in individuals on long-term sick leave [4–7] and improving cognitive and emotional functioning will enhance the ability to stay focused, process and remember information, and shift focus when required because individuals have increased mental resources and capacity [7]. Emotional functioning refers to our ability to regulate and label our emotions enabling us to influence and direct our attention away from negative emotions and biases resulting in better coping [10]. The benefits of improved cognitive and emotional functioning are better flexibility, better regulation of our emotions and experiences, and increased quality of life [8–10]. Efforts have been made to better understand cognitive as well as emotional functioning in long-term sick-listed individuals participating in occupational rehabilitation [4, 7, 11–13]. In Norway, the occupational rehabilitation programmes are designed to facilitate return to work (RTW) through physical and psychological empowerment and communication with the employer.

We have recently reported that focused and sustained attention improved more than memory, executive function and emotion recognition during occupational rehabilitation [7]. The next step is to investigate whether baseline scores and change scores during rehabilitation in cognitive and emotional functioning, such as sustained attention and emotion recognition, are associated with a higher probability of RTW when the duration of sick leave before enrolment in the programme is taken into account. If such associations are present, the treatment success of occupational rehabilitation may depend, at least partly, on improving cognitive functioning [7] and cognitive beliefs related to work through cognitive therapy [14].

The treatment components in occupational rehabilitation programmes have a cognitive behavioural approach and consist of an assessment of the work and health situation, physical activity, individual consultations, and collaboration with the workplace [15, 16]. The cognitive approach draws on principles and interventions from evidence-based psychological treatments such as cognitive therapy, acceptance and commitment therapy and motivational interviewing [17–19]. Psychological interventions are the most common form of treatment for mental health problems such as anxiety, depression, pain and stress [20], which are prevalent in the patient groups being referred to occupational rehabilitation [15, 21]. Psychological interventions have also shown to improve functional outcomes such as physical functioning, coping with pain and fatigue [20, 22] and RTW [18]. However, functional changes seem to occur to a lesser extent compared to changes in cognition and behaviour [20]. The effect of using a cognitive approach, together with other treatment components, has shown that work participation increased for patients attending a long inpatient programme compared to a six week outpatient programme with two weekly hours of treatment [16], while a short inpatient programme, with the same treatment components as the long, was also compared to the outpatient programme but showed no superior effects on work participation [17].

Given the documentation of cognitive impairments in individuals on sick leave [5, 6, 23–26], the association between cognitive functioning and RTW has not received sufficient attention. Besides, other studies have also reported that impaired cognitive and executive functioning have been found to negatively affect occupational status [27–29]. The present study sought to overcome some of the methodological limitations in previous studies investigating the relationship between cognition and RTW. These studies did not obtain register-based sick leave [30, 31], generally had small sample sizes when investigating RTW [31], failed to include emotional tests [25] and interventions were not provided in a systematic manner [5, 6]. In the current study, objective cognitive and emotional tests were administered, health-related benefits status up to one year after rehabilitation, based on register data, were obtained, and all patients participated in occupational rehabilitation. It was expected that attention would be associated with the number of days on health-related benefits in the year following rehabilitation as specific improvements in functioning related to attention is likely to occur during the rehabilitation programmes. Thus, the aim of the study was to investigate the association between cognitive and emotional functioning and RTW, within two groups of patients characterised by different durations of sick leave before participation in occupational rehabilitation.

## Methods

### Participants

In total, 317 individuals completing either inpatient or outpatient occupational rehabilitation were recruited from four clinics. Those that had received health-related benefits for the full year before rehabilitation comprised the work assessment allowance and disability pension (WAA) group and those receiving health-related benefits for less than a year comprised the sick leave (SL) group. This split was decided upon because, in the Norwegian health-related benefit system, there is a natural step from sick leave benefits after one year, where 100% of wages are compensated, to work assessment allowance benefits, where 66% of wages are compensated. Eight participants did not receive health-related benefits at the time of inclusion in the study but were on full time (inpatient) or part time (outpatient) sick leave during rehabilitation. The majority of patients had diagnoses in the categories *M*, diseases of the musculoskeletal system and connective tissue (53%), *F*, mental and behavioural disorders (27%), or, *G*, disease of the nervous system (8%) within ICD-10 [32]. Individuals with a history of head injury or in the process of applying for full disability pension were excluded from the study.

### Study Design

This study was a multicentre prospective cohort study involving four rehabilitation clinics. All participants were followed for 12 months with register data on the health-related benefit status provided by the the Norwegian Labour and Welfare Administration. The participants completed cognitive and emotional tests and questionnaires on the topics of work and health pre and post rehabilitation. That is, on the first, second or third day after arrival at the rehabilitation clinic (baseline), and to enable the calculation of change scores the participants completed a second assessment one to three days before the end of rehabilitation (change score). All assessments took place in a quiet room at each clinic and completion of the tests and questionnaires took approximately 1 h and 30 min at each assessment. Three research assistants, who all took online training provided by Cambridge Cognition in administering the Cambridge Neuropsychological Test Automated Battery (CANTAB), and the first author (TJ), having extensive training in neuropsychological administration, were responsible for all data collection.

### Intervention

The duration of the rehabilitation programmes varied between the four clinics from three to 12 weeks. The clinics had similar treatment components which included physical activity adjusted according to patients’ capacity applying endurance and resistance exercises, cognitive behaviour treatment components based on principles from cognitive behaviour therapy focusing on work and health issues, and when deemed appropriate, collaboration with the workplace, the patients’ general practitioners, and the social security office. The majority of patients made a written plan during rehabilitation specifying the steps needed to RTW. Patients were followed up individually and in groups by an interdisciplinary team consisting of, but not limited to, a physician, physiotherapist, sports pedagogue, psychologist, work consultant/coach and nurse/psychiatric nurse.

### Health-Related Benefits System

In Norway, medically certified sick leave is granted for a maximum of 52 weeks with 100% compensation of which the employer is responsible for economic compensation during the first 16 days, and after that, the Norwegian Labour and Welfare Administration. If long term benefits are required after 52 weeks it is possible to apply for work assessment allowance of which 66% of the wage is compensated. This can be granted for a maximum of 3 years and during this period or after, disability pension may be granted. All benefits can be granted in combination with partial work participation and are commonly named sick leave benefits, work assessment allowance benefits and disability benefits.

### Materials

More details about the cognitive and emotional tests and the work and health related questionnaires are available from Johansen et al. [7].

### Tests on Cognitive and Emotional Functions

A battery of eight cognitive and emotional tests from the CANTAB was administered to cover a broad range of functions. The following tests were administered: Simple Reaction Time, Choice Reaction Time, Rapid Visual Information Processing, Spatial Working Memory, Spatial Recognition Memory, Stockings of Cambridge (a version of the Tower of London task measuring executive planning), Intra-Extra Dimensional Set Shift, Emotion Recognition Task. All tests were administered on a touch-sensitive computer screen. The administration of the tests was counterbalanced in two orders so that each participant experienced each order once. This was carried out to avoid the effects of order which could potentially influence the performance.

### Work and Health Questionnaires

The following questionnaires and single-item questions were administered: Work ability measured by one item comparing current work ability with lifetime best [33]; Expectation to RTW based on one item asking about when the participant expected to RTW [34]; Return to Work Self-Efficacy (RTWSE–19) [35, 36]; Subjective Health Complaints (SHC) inventory [37]; Theoretically Originated Measure of the Cognitive Activation Theory of Stress (TOMCATS) [38]; Fear Avoidance Beliefs Questionnaire (FABQ) [39]; Hospital Anxiety and Depression Scale (HADS) [40].

### Statistical Analysis

SPSS version 25 was used to analyse the data (SPSS Inc., 2019). As described under participants, the sample was split into two groups based on the individuals’ health-related benefit status in the year before rehabilitation. The cognitive and emotional distribution of baseline and change scores were both graphically and descriptively examined in terms of skewness and outliers. It was decided to remove extreme latencies and error rates, which were considered subtle and clearly distinguishable from the rest [41]. In total, six outliers were removed. Between-group differences at baseline were examined for demographic, work and health characteristics and baseline performance in cognitive and emotional functioning using independent samples t-tests. Gender, education and expectation to RTW were subjected to chi-square analysis. The two groups were separately subjected to multiple linear regression analysis. The predictor variables were the tests within the cognitive domains attention, memory, executive function, and emotion. The outcome variable was measured using register data on health-related benefits one year from the second assessment and was the accumulated number of days on either sick leave, work assessment allowance or disability pension. The number of days was counted from the second assessment to take into account the difference in duration of the rehabilitation programmes between the four clinics. Graded benefits were converted to full days. This ensured that all health-related benefit days were counted from the same time point for all participants. The analyses were split in two, first using baseline cognitive and emotional scores as predictors and secondly using the change scores in cognitive and emotional performance as predictors. Prior to the multiple regression analysis, the association between each of the cognitive and emotional predictors (baseline scores and change scores) and the dependent variable was separately examined in the two groups by bivariate linear regression analyses. Three multiple regression models were subsequently created.

Model 1: Predictors associated with the dependent variable at a statistically significant level of p < 0.20 in the bivariate analyses were further analysed in multiple regression analyses controlling for age, gender and education, separately for each cognitive and emotional domain (see Tables 3–6). Model 2: Same as model 1 but adding the variables SHC pseudoneurology and SHC musculoskeletal pain. Model 3: Same as model 1 but adding the variables HADS anxiety and HADS depression. The independent variables included in the three models were separately checked for multicollinearity in the WAA and SL group by the variance inflation factor (VIF), where values > 5 indicate multicollinearity [42]. Statistical significance was accepted with a two-tailed p-value of ≤ 0.05.

## Results

There were no group differences at baseline in age and education, while the number of female participants was higher in the WAA compared to the SL group (Table 1). Participants in the SL group had expectations about faster RTW compared to the WAA group. For the work variables, the SL group compared to the WAA group reported higher work ability and higher RTW self-efficacy for the factors “meeting job demands” and “modifying job tasks”. For the health variables, the SL group showed better coping and lower scores on the SHC pseudoneurology, TOMCATS hopelessness, FABQ for work and physical activity, and HADS depression.

**Table 1:** Demographic, work and health characteristics at baseline

	Work assessment allowance and disability pension (n = 181)		Sick leave (n = 136)		Statistics	
Variable	Mean	SD	Mean	SD	t (df)^#^	p-value
Age	45.3	9.8	44.3	9.7	0.936 (315)	0.350
Number of days on health-related benefits one year after rehabilitation	263.2	90.1	15.5	14.5	Not applicable	
Work ability (0–10; 10 = best work ability)	3.0	2.1	4.8	2.2	− 6.997 (291)	0.000
RTWSE-19						
Meeting job demands (1–70; 70 = highest SE)	28.4	17.3	40.7	17.8	− 5.670 (266)	0.000
Modifying job tasks (1–60; 60 = highest SE)	26.4	12.9	31.3	13.7	− 2.975 (263)	0.003
Communicating needs (1–60; 60 = highest SE)	34.9	14.8	38.2	14.2	− 1.826 (269)	0.069
SHC						
Pseudoneurology (0–21; 21 = most complaints)	7.6	4.1	6.7	4.2	1.977 (289)	0.049
Musculoskeletal pain (0–24; 24 = most complaints)	10.3	4.9	9.6	5.2	1.103 (286)	0.271
TOMCATS						
Coping (1–4; 1 = best coping))	2.1	0.6	1.9	0.6	2.443 (285)	0.015
Hopelessness (1–12; 1 = most hopelessness)	8.8	1.9	9.4	1.9	− 2.737 (286)	0.007
Helplessness (1–12; 1 = most helplessness)	9.5	2.1	9.7	1.9	− 1.148 (284)	0.252
FABQ						
Work (0–42; 0 = no fear avoidance)	21.9	11.4	18.8	11.2	2.258 (266)	0.025
Physical activity (0–24; 0 = no fear avoidance)	9.7	6.1	8.2	5.9	2.078 (270)	0.039
HADS						
Anxiety (0–21; 0 = no anxiety)	8.6	4.1	7.9	4.6	1.265 (280)	0.207
Depression (0–21; 0 = no depression)	6.8	3.9	5.7	3.8	2.232 (280)	0.026

Variable	n	%	n	%	Χ^2^ (df)^#^	
Gender						
Female	131	72	78	57	7.802 (1)	0.005
Male	50	28	58	43		
Education						
Elementary	23	13	16	12	0.426 (2)	0.808
Secondary	73	43	62	46		
Higher	75	44	56	42		
Expectation to return to work						
Within 3 months	58	37	99	79	49.039 (1)	0.000
More than 3 months	97	63	26	21		

*SD* standard deviation, *Χ*^2^ chi-square statistic, *RTWSE-19* return-to-work self-efficacy, *SHC* subjective health complaints inventory, *TOMCATS* theoretically originated measure of the cognitive activation theory of stress, *FABQ* fear avoidance beliefs questionnaire, *HADS* hospital anxiety and depression scale

^#^Not all participants responded

Overall, the SL participants performed better on most of the cognitive and emotional tests compared to the WAA group, where significant group differences were found in focused attention on the simple and choice reaction time tests and in executive function on the stockings of Cambridge task (Table 2).

**Table 2:** Cognitive and emotional performance at baseline

Variables	Work assessment allowance and disability pension (n = 179)		Sick leave (n = 135)		Statistics	
	Mean	SD	Mean	SD	t (df)	p-value
Attention						
Simple reaction time						
Reaction time (milliseconds)	264.8	73.1	248.5	39.8	2.334 (312)	0.020
Choice reaction time						
Reaction time (milliseconds)	329.6	74.0	313.2	49.8	2.214 (310)	0.028
Rapid visual information processing						
Latency (milliseconds)	411.9	89.2	406.1	84.7	0.574 (305)	0.567
Probability of hit	0.60	0.20	0.62	0.15	− 0.990 (306)	0.323
Memory						
Spatial working memory						
Total between errors	13.7	10.0	11.9	9.8	1.573 (313)	0.117
Spatial recognition memory						
Response time (milliseconds)	2729.4	1032.7	2716.5	767.3	0.121 (311)	0.904
Total correct (%)	79.5	10.2	81.4	9.9	− 1.669 (313)	0.096
Executive function						
Stockings of Cambridge						
Choice duration (milliseconds)	4007.4	1752.5	4279.0	2210.7	− 1.208 (309)	0.228
Total correct	8.6	2.1	9.1	2.0	− 2.128 (313)	0.034
Intra-extra dimensional set shift						
Trials extradimensional shift stage	10.1	9.3	8.5	8.8	1.502 (312)	0.134
Emotion recognition						
Emotion recognition task						
Total correct (%)	59.0	10.1	58.2	10.5	0.646 (312)	0.519

Sustained attention and executive function were associated with the number of days on health-related benefits in the year after rehabilitation for the WAA group (Table 3) and working memory and executive function for the SL group (Table 4). Thus, these variables were separately included for each group, as baseline predictors and change score predictors, in the multiple regression analysis.

**Table 3:** Bivariate linear regression analysis for the work assessment allowance and disability pension group using baseline and change scores from cognitive and emotional tests to examine the association with number of days on health-related benefits up to one year after rehabilitation

	Work assessment allowance and disability pension (n = 165)					
	Baseline predictors			Change score predictors		
	β, Beta	95% CI	p	β, Beta	95% CI	p
Attention						
SRT reaction time (s)	69.737	− 112.896/252.370	0.452	− 100.287	− 419.546/218.973	0.536
CRT reaction time (s	− 15.602	− 197.519/166.615	0.866	99.511	− 123.504/322.526	0.379
RVP latency (s)	**− 170.972**	**− 321.472/− 20.471**	**0.026**	**− 195.375**	**− 357.519/− 33.231**	**0.019**
RVP probability of hits	− 13.038	− 82.052/55.976	0.710	− 8.523	− 112.246/95.201	0.871
Memory						
SWM total errors	− 0.804	− 2.127/.518	0.232	− 0.450	− 2.211/1.311	0.614
SRM latency (s)	− 8.311	− 21.119/.4.496	0.202	6.629	− 9.582/22.839	0.421
SRM total correct (%)	0.357	− .943/1.658	0.589	0.149	− 1.067/1.366	0.809
Executive function						
SOC choice duration (s)	3.969	− 3.644/11.581	0.305	**7.970**	**− 0.664/16.605**	**0.070**
SOC total correct	1.080	− 5.259/7.419	0.737	− 2.976	− 10.360/4.408	0.427
EDS trials	− 0.454	− 1.881/.973	0.531	− 0.082	− 1.835/1.672	0.927
Emotion recognition						
ERT total correct (%)	− 0.483	− 1.792/0.827	0.468	− 0.545	− 2.593/1.503	0.600

Bold values denote statistical significance at the p < 0.20 level

*SRT* simple reaction time, *CRT* choice reaction time, *RVP* rapid visual information processing, *SWM* spatial working memory, *SRM* spatial recognition memory, *SOC* stockings of Cambridge, *EDS* intra-extra dimensional set shift, *ERT* emotion recognition task

**Table 4:** Bivariate linear regression analysis for the sick leave group using baseline and change scores from cognitive and emotional tests to examine the association with number of days on health-related benefits up to one year after rehabilitation

	Sick leave (n = 132)					
	Baseline predictors			Change score predictors		
	β, Beta	95% CI	p	β, Beta	95% CI	p
Attention						
SRT reaction time (s)	− 24.431	− 86.837/37.975	0.440	− 26.692	− 96.540/43.155	0.451
CRT reaction time (s)	− 1.482	− 51.372/48.408	0.953	− 20.815	− 89.295/47.665	0.548
RVP latency (s)	− 6.972	− 36.792/22.848	0.644	− 14.310	− 45.089/16.470	0.359
RVP probability of hits	− 5.042	− 21.879/11.075	0.518	6.479	− 12.030/25.527	0.478
Memory						
SWM total errors	**0.290**	**0.040/0.540**	**0.023**	**0.234**	**− 0.125/0.593**	**0.200**
SRM latency (s)	− 0.560	− 3.849/2.729	0.737	0.777	− 2.962/4.517	0.681
SRM total correct (%)	− 0.133	− 0.385/0.118	0.296	0.014	− 0.217/0.245	0.903
Executive function						
SOC choice duration (s)	**− 0.903**	**− 2.028/.222**	**0.115**	**− 1.069**	**− 2.461/.323**	**0.131**
SOC total correct	− 0.754	− .2.004/0.497	0.235	0.194	− 1.271/1.660	0.793
EDS trials	0.030	− 0.255/0.314	0.837	0.094	− 0.208/0.396	0.538
Emotion recognition						
ERT total correct (%)	− 0.087	− 0.326/0.152	0.474	0.035	− 0.398/0.469	0.874

Bold values denote statistical significance at the p < 0.20 level

*SRT* simple reaction time, *CRT* choice reaction time, *RVP* rapid visual information processing, *SWM* spatial working memory, *SRM* spatial recognition memory, *SOC* stockings of Cambridge, *EDS* intra-extra dimensional set shift, *ERT* emotion recognition task

### Cognitive Baseline and Change Score Predictors and Number of Days on Health-Related Benefits in the Work Assessment Allowance and Disability Pension Group

Regression model 1 indicated that latency on the rapid visual information processing test was significant both at baseline (t (163) =  − 2.574, p = 0.011) and as change score (t (150) =  − 2.527, p = 0.013) (Table 5). Latency on the rapid visual information processing test remained significant in models 2 and 3 after controlling for SHC pseudoneuroloy and musculoskeletal pain and HADS anxiety and depression respectively. In model 3, the change score for HADS depression was also significant (p = 0.019). For the domain executive function, the change score for HADS depression was significant in model 3 (p = 0.044) (Table 5). These results did not change when the same analyses were run including the outliers.

**Table 5:** Multiple linear regression analysis for the work assessment allowance and disability pension group using significant baseline and change score predictors together with age, gender and education to examine the association with number of days on health-related benefits up to one year after rehabilitation

	Work assessment allowance and disability pension (n = 165)					
	Baseline predictors			Change score predictors		
	β, Beta	95% CI	p	β, Beta	95% CI	p
Attention model 1				Attention		
RVP latency (s)	**− 202.936**	**− 358.653/− 47.219**	**0.011**	**− 205.591**	**− 366.377/− 44.806**	**0.013**
Attention and SHC model 2						
RVP latency (s)	**− 184.489**	**− 350.012/− 18.966**	**0.029**	**− 207.482**	**− 388.808/− 26.157**	**0.025**
SHC pseudoneurology	− 0.727	− 4.550/3.097	0.708	− 0.892	− 6.603/4.819	0.758
SHC musculoskeletal pain	− 2.067	− 5.379/1.245	0.219	− 2.374	− 7.257/2.508	0.337
Attention and HADS model 3						
RVP latency (s)	**− 206.905**	**− 371.773/− 42.036**	**0.014**	**− 240.993**	**− 413.977/− 68.009**	**0.007**
HADS anxiety	− 1.236	− 5.818/3.347	0.595	0.342	− 5.841/6.525	0.913
HADS depression	0.277	− 4.435/4.990	0.116	**− 6.906**	**− 12.658/− 1.154**	**0.019**
Executive function model 1						
SOC choice duration (s)				7.419	− 1.195/16.032	0.091
Executive function and SHC model 2						
SOC choice duration (s)				6.680	− 2.436/15.795	0.149
SHC pseudoneurology				− 0.156	− 5.843/5.531	0.957
SHC musculoskeletal pain				− 4.015	− 8.730/0.700	0.094
Executive function and HADS model 3						
SOC choice duration (s)				6.325	− 2.835/15.485	0.174
HADS anxiety				2.069	− 4.151/8.288	0.512
HADS depression				**− 5.814**	**− 11.481/− 0.148**	**0.044**

Bold values denote statistical significance at the p < 0.05 level

### Cognitive Baseline and Change Score Predictors and the Number of Days on Health-Related Benefits in the Sick Leave Group

Errors on the spatial working memory test was significant at baseline in regression model 1 (t (131) = 2.067, p = 0.041) and 2 (t (122) = 2.533, p = 0.013) (Table 6). For the domain executive function, choice duration on the stockings of Cambridge test at baseline was significant in model 3 (t (121) = -2.051, p = 0.043). The results did not change when the same analyses were run including the outliers.

**Table 6:** Multiple linear regression analysis for the sick leave group using significant baseline and change score predictors together with age, gender and education to examine the association with number of days on health-related benefits up to one year after rehabilitation

	Sick leave (n = 132)					
	Baseline predictors			Change score predictors		
	β, Beta	95% CI	p	β, Beta	95% CI	p
Memory						
SWM total errors	**0.288**	**0.012/0.564**	**0.041**	0.238	− 0.125/0.601	0.197
Memory and SHC Model 2						
SWM total errors	**0.384**	**0.084/0.684**	**0.013**	0.293	− 0.147/0.734	0.189
SHC pseudoneurology	0.418	− 0.275/1.111	0.235	− 0.126	− 1.289/1.037	0.830
SHC musculoskeletal pain	0.087	− 0.488/0.661	0.766	0.011	− 0.896/0.918	0.981
Memory and HADS Model 3						
SWM total errors	0.286	− 0.005/0.576	0.054	0.252	− 0.158/0.663	0.226
HADS anxiety	0.612	− 0.200/1.424	0.138	− 0.485	− 1.695/0.724	0.428
HADS depression	0.073	− 0.903/1.048	0.883	0.036	− 1.084/1.157	0.949
Executive function model 1						
SOC choice duration (s)	− 1.105	− 2.277/0.068	0.065	− 1.191	− 2.608/0.226	0.099
Executive function and SHC Model 2						
SOC choice duration (s)	− 1.215	− 2.436/0.006	0.051	− 1.417	− 3.089/0.255	0.096
SHC pseudoneurology	0.339	− 0.360/1.037	0.339	− 0.247	− 1.414/0.919	0.675
SHC musculoskeletal pain	0.210	− 0.380/0.800	0.483	− 0.069	− 0.970/0.832	0.879
Executive function and HADS Model 3						
SOC choice duration (s)	**− 1.261**	**− 2.479/− 0.043**	**0.043**	− 1.357	− 2.956/0.242	0.095
HADS anxiety	0.543	− 0.273/1.359	0.190	− 0.580	− 1.781/0.621	0.340
HADS depression	0.098	− 0.881/1.077	0.843	0.131	− .994/1.256	0.817

Bold values denote statistical significance at the p < 0.05 level

The VIF of the independent variables in the three models for the WAA and SL group were all below 2.0, indicating no multicollinearity.

## Discussion

The association between cognitive and emotional functioning and RTW in employees on health-related benefits is under-studied. We investigated this relationship in work assessment allowance, disability pension and sick leave groups participating in occupational rehabilitation. Individuals in the WAA group had been on health-related benefits for the whole year before entering the rehabilitation programme, while the SL group had been on benefits for less than a year. Our results indicated that baseline and change scores from the start to the end of rehabilitation for sustained attention in the WAA group and baseline scores for working memory in the SL group were associated with fewer number of health-related benefit days in the year after rehabilitation. That is, better functional status in sustained attention and working memory at baseline, and the greater the improvement in sustained attention during rehabilitation, the fewer days on health-related benefits are expected. The association seemed strongest in the WAA group, as the effect of sustained attention remained even after controlling separately for SHC pseudoneuroloy and musculoskeletal pain and HADS anxiety and depression. In the SL group, the working memory baseline association remained when controlling for SHC pseudoneuroloy and musculoskeletal pain. In the WAA group, change scores for depression showed an association with days on health-related benefits, and in the SL group, baseline scores for executive function also showed an association, albeit difficult to interpret. Therefore, in the following, we focus on the most robust results and discuss the cognitive aspects related to work for sustained attention and working memory. The WAA and SL group differed in cognitive performance at baseline, with the former scoring worse in focused attention and executive function. On the work variables, the WAA group reported lower work ability and RTW self-efficacy compared to the SL group. They also had lower expectations about RTW, where the majority reported that it would take more than three months to RTW. The WAA group reported lower health status compared to the SL group as they scored higher in SHC pseudoneurology symptoms, hopelessness, fear avoidance for work and physical activity, depression and worse on coping.

In line with the present findings, a recent study reported an association between subjective cognitive complaints and sickness absence in a specific occupational group [43]. While the current study used objective measures of cognition through computerised testing, it may be plausible that both objective and subjective assessments of cognitive impairments could be associated with sickness absence and RTW. Studies using sick leave status based on self-report have either not investigated the association between objective assessments of cognition and RTW [25, 44] or failed to find an association despite substantial improvement in memory and attention and an increase in RTW two years after a workplace intervention [30, 31]. Official data from registries, as collected in the present study, is often preferred due to the longitudinal and validated nature of data, which is often hard to obtain through self-report [45].

Several potential mechanisms may explain an association between sustained attention, working memory and fewer days on health-related benefits. While supposed to be capacity limited, sustained attention is needed to keep us continuously focused for more than a few seconds while ignoring competing or distracting information. Working memory represents a cognitive function that retains information over the short term and enables us to act on that information. As both functions seem to have capacity limitations and depend on each other in selecting and storing information, our attentional system must select the most relevant information to be stored in working memory [46, for a detailed review]. Working memory and attention are also dependent on the control of the executive functions inhibition, updating, and shifting of attention [47]. These are key factors in attention and executive control [48, 49]. We know that engaging in specific goal-related and repetitive tasks are important in any work situation [50], and these tasks require working memory to be constantly updated throughout the day with the support of sustained attention. This is based on the argument that being in work helps maintain both attention and working memory to operate efficiently, because work can be seen as a training arena for cognitive functions [2]. This gives support to the hypothesis of «use it or lose it» [2, 51]. Our ability to stay focused is more likely to increase if the demands at work on sustained attention and working memory are high [3, 50] and when we perform complex tasks either at home or in work [2]. Therefore, occupational rehabilitation [7, 13], physical activity [52], better emotion regulation [9, 10] or attention bias modification training [53] also improve cognitive and emotional functions and seem likely to pave the way for better performances at work.

## Clinical Implications

The current study adds further knowledge about occupational rehabilitation and presents an association between cognition and RTW in the WAA and SL group. Previous findings from our group have demonstrated that focused and sustained attention and working memory improve more than executive function and emotion recognition during rehabilitation [7, 13]. Although it cannot be elucidated at this stage which interventions in the rehabilitation programme improve attention and working memory, it can be claimed that the combined effects of all treatment components [54], such as physical activity, cognitive approach, collaboration with the workplace and following an RTW plan, improve certain cognitive functions more than others.

The present findings emphasise the importance of assessing cognitive functioning in different patient groups based on the length of sick leave. If such assessments are not conducted, clinicians are left with only self-reported assessments of work and health and may fail to meet the goal of a holistic [55] and comprehensive assessment [4]. The implications for clinical practice revolve around the issue of identifying those individuals displaying cognitive impairments at baseline while at the same time investigating both their benefit and work status. This adds to the debate about when different work-related interventions could be applied and for whom [56]. It could be argued that WAA individuals may require more specific interventions related to the cognitive function sustained attention, while SL individuals may benefit from working memory interventions. This postulation is worth following up as sick leave is associated with a deterioration in health and quality of life [57, 58], but also the fact that improvements in attention are associated with better work ability and a reduction in subjective health complaints [7]. The treatment success of occupational rehabilitation may depend, at least partly, on improving cognitive functioning, specifically sustained attention, to increase the chances of RTW for individuals having been away from work for more than a year [7, 14].

Specific cognitive training may improve certain cognitive functions, and this has been carried out for chronic pain [59], depression, [53] and occupational rehabilitation patients [12]. However, these training methods have to be carefully selected bearing in mind that working memory training does not seem transferable to cognitive abilities required at work or in everyday life [60]. Currently, it seems more fruitful to develop training programmes that show an effect on work-related factors [61] to improve work-related working memory and sustained attention. The promising attention bias modification task [53] could be adapted to work settings and is a fruitful avenue to pursue. Such training, in combination with the cognitive approach, physical activity and collaboration with the workplace, may be worth piloting in collaboration between researchers and clinicians. These suggestions may result in occupational rehabilitation programmes becoming more individually tailored according to benefit status, while still maintaining the group-based approach in most interventions.

## Study Limitations

Recruiting patients from four different clinics could be a potential confounder in the study. Despite that all patients received the same treatment components in occupational rehabilitation, differences in procedures, intervention dosage and alliances with the patients at the four clinics could not be accounted for. Only the first item in the work ability index (current work ability compared with the lifetime best) was used as opposed to the entire measure of seven items [62], and we cannot claim that we measured the whole concept of work ability. The rationale for using one item, as opposed to the entire measure, was due to its predictive value on RTW [33] and the fact that not all items were applicable to this patient group. Another limitation of our study is that the findings cannot explain which treatment components in the rehabilitation programme positively affected sustained attention and working memory which were associated with fewer health-related benefit days in the year after rehabilitation. It can only be assumed that the combination of all interventions contributed to the association between cognition and RTW.

## Conclusion

This study has demonstrated that better sustained attention and working memory are associated with fewer health-related benefit days in the year following rehabilitation. These results showed that baseline and change scores in cognitive performance during occupational rehabilitation could be an indicator of future days on health-related benefits after rehabilitation. Sustained attention and working memory are interlinked and important functions to keep intact to enable performances in most occupations. The quality of occupational rehabilitation programmes could be enhanced if work-related sustained attention and working memory interventions are respectively targeted in individuals on sick leave or work assessment allowance and disability pension.
